# Abnormal saline: the unphysiological bag of brine

**DOI:** 10.1186/s13054-014-0673-z

**Published:** 2014-11-28

**Authors:** Mourad H Senussi

**Affiliations:** Respiratory Institute, Cleveland Clinic, 9500 Euclid Avenue, Cleveland, OH 44195 USA

Duburcq and colleagues, in their article published in *Critical Care*, showed improved oxygenation, hemodynamic parameters, and microvascular reactivity in an experimental porcine endotoxic shock model with use of hypertonic sodium lactate 11.2% [1]. We applaud the authors for embarking on the arduous quest for the ideal resuscitative fluid, which has historically been fraught with shortcomings.

The control group in this experimental model was subjected to an infusion of 0.9% normal saline. Normal saline has become almost ubiquitous in patient care. More evidence is emerging that this so-called lifeblood, as unphysiological as it is, may be causing undue harm to our patients. A 0.9% normal saline infusion has been demonstrated to cause decreased renal blood flow velocity and renal cortical tissue perfusion in healthy volunteers [2]. Abnormal saline was also associated with increased incidence of acute kidney injury and need for renal replacement therapy in a cohort of critically ill patients [3] and had a higher in-hospital mortality rate than more balanced salt solutions [4]. The significant chloride content of normal saline has been implicated in its adverse effect on acid–base status. It has become more evident that the use of balanced salt solutions may be associated with reduced mortality in the septic population [4,5].

Given the mounting evidence against the use of normal saline, I believe it would be better served if, firstly, the normal saline arm is replaced with one with a balanced salt solution. Secondly, I suggest the experimental model should include a true control arm that did not receive any resuscitative fluids.
